# Development and validation of a predictive model for early diagnosis of neonatal acute respiratory distress syndrome based on the Montreux definition

**DOI:** 10.3389/fped.2023.1276915

**Published:** 2023-11-02

**Authors:** Leilei Shen, Na Cai, Shaoyou Wan, Sheng Chen

**Affiliations:** Department of Pediatrics, The First Affiliated Hospital of Army Medical University, Chongqing, China

**Keywords:** neonate, acute respiratory distress syndrome, Montreux definition, prediction model, early diagnosis

## Abstract

**Objective:**

Based on the Montreux definition, we aim to develop and validate a predictive model for the early diagnosis of neonatal acute respiratory distress syndrome (ARDS).

**Methods:**

A retrospective analysis of clinical data on 198 neonates with respiratory distress from January 2018 to January 2022 was conducted. Neonates meeting Montreux definition were classified as ARDS group (*n* = 79), while the rest were non-ARDS group (*n* = 119). Univariate analysis identified indicators for neonatal ARDS, followed by logistic regression to construct a predictive model for early diagnosis. The ability of predictors and models to predict neonatal ARDS was evaluated using area under the curve (AUC), and model performance was estimated through bootstrap resampling.

**Results:**

Maternal prenatal fever, abnormal fetal heart beat, meconium-stained amniotic fluid (MSAF), white blood cell (WBC), absolute neutrophil count (ANC), neutrophil percentage (NE%), platelet count (PLT), C-reactive protein (CRP), procalcitonin (PCT), creatine kinase (CK), activated partial thromboplastin time (APTT), serum calcium (Ca) and sodium (Na)exhibited significant differences between the ARDS group and the non-ARDS group (*P *< 0.05). MSAF (OR=5.037; 95% CI: 1.523–16.657; *P *< 0.05), ANC (OR = 1.324; 95% CI: 1.172–1.495; *P *< 0.05), PLT (OR = 0.979; 95% CI: 0.971–0.986; *P *< 0.05), Ca (OR = 0.020; 95% CI: 0.004–0.088; *P *< 0.05) emerged as independent risk factors for the development of ARDS. The respective AUC values for MSAF, ANC, PLT, Ca, and the combined prediction models were 0.606, 0.691, 0.808, 0.761 and 0.931. Internal validation showed that the C-index for the model was 0.931.

**Conclusions:**

Early application of the model combining MSAF, ANC, PLT and Ca may have a good predictive effect on the early diagnosis of neonatal ARDS.

## 1. Introduction

Neonatal acute respiratory distress syndrome (ARDS) is a common and life-threatening respiratory condition frequently encountered in neonatal intensive care units (NICUs). It is a major cause of neonatal mortality and disability, with a mortality rate of approximately 20% (1). It represents an acute pulmonary inflammatory disease caused by entrapment or extrapulmonary factors. The primary pathological characteristics include pulmonary inflammatory cell infiltration, alveolar epithelial and capillary endothelial cell injury, disruption of alveolar epithelial barrier function, and increased vascular endothelial permeability (2). Despite extensive research on ARDS in children and adults, the etiology, clinical manifestations, treatment and prognosis of ARDS in neonates may differ due to distinct lung development and immune function (3). Therefore, the diagnostic criteria used for ARDS in children and adults (Berlin Definition) (4) and PALICC Definition (5) cannot be applied to neonates (6). In 2017, an international, multi-center and multidisciplinary team in Montreux, Switzerland, established the first diagnostic criteria for neonatal ARDS (Montreux definition) based on extensive evidence-based medical research (6). The “Montreux definition” continue with the previous diagnostic criteria for ARDS in children and adults, and fully take into account the special physiological characteristics of newborns, such as small tidal volume, high respiratory rate, small airways prone to increased respiratory resistance, etc., emphasizing the predisposing factors diversity, and can be distinguished from neonatal respiratory distress syndrome (NRDS), transient tachypnea of the newborn (TTN), and surfactant-related genetic defect diseases, so it is more suitable for newborns. The implementation of this standard has had a significant positive impact on the diagnosis, treatment and research of neonatal ARDS.

Due to the complex etiology and pathogenesis of neonatal ARDS, there is a lack of specific therapeutic approaches for this condition. Early identification and proactive early intervention play a critical role in improving the prognosis and reducing the mortality in neonates with ARDS. However, the prevailing risk prediction models for ARDS, such as the lung injury prediction score (LIPS) model (7), the N-gram model (8), the ARDS early warning system (9) and the ARDS early risk prediction model (10), were primarily developed for adult populations and are therefore unsuitable for neonates. Therefore, the development of a predictive model specifically tailored to neonatal ARDS has significant clinical implications for the timely detection and management of this condition. In this study, we retrospectively analyzed clinical data on neonatal ARDS from our NICU, with the aim of developing an early diagnostic model for neonatal ARDS, thus providing a basis for early therapeutic interventions in affected infants.

## 2. Methods

### 2.1. Study subjects

This retrospective cohort study was conducted in the NICU of the First Affiliated Hospital of the Army Medical University of China. The study analyzed the clinical data of 198 newborns admitted between January 2018 and January 2022 with respiratory distress as the main clinical manifestation, either starting on the day of birth or admitted with other diseases and developing symptoms of respiratory distress during hospitalization. Peripheral blood was collected from these newborns within 24 h of the onset of respiratory distress for laboratory testing. The diagnosis of neonatal ARDS was made using the “Montreux definition”, which resulted in 79 cases being assigned to the ARDS group, while the remaining 119 cases were assigned to the non-ARDS group. The exclusion criteria were as follows: (1) Dyspnea caused by congenital malformations (pulmonary adenoma malformation, diaphragmatic hernia, etc.), NRDS, and TTN that have different pathophysiological basis from ARDS; (2) Genetic defects related to pulmonary surfactant cause clinical manifestations similar to ARDS.

This study was approved by the Ethics and research Committee of the First Affiliated Hospital, Army Medical University, Chongqing, China (KY202249), and all research procedures were conducted according to the principles of the Declaration of Helsinki.

### 2.2. Clinical definitions

The Montreux definition encompass the following aspects: (1) acute onset (i.e., within one week) from a known or suspected clinical insult; (2) exclusion of respiratory distress resulting from NRDS, TTN, or congenital anomalies; (3) lung imaging displaying bilateral diffuse irregularities with reduced translucency, exudate, or opacities not explained by additional factors such as localized effusion, pulmonary atelectasis, NRDS, TTN, or congenital anomalies; (4) absence of congenital heart disease explaining the oedema (this includes ductus arteriosus with pulmonary overflow if no acute pulmonary haemorrhage exists). Echocardiography is needed to verify the origin of oedema; and (5) oxygenation deficit quantified as Oxygenation Index (OI): Mild ARDS: 4 ≤ OI < 8, Moderate ARDS: 8 ≤ OI < 16, Severe ARDS: OI ≥ 16. All five criteria must be met for the diagnosis of neonatal ARDS.

Neonatal sepsis was defined as the growth of at least a single pathogen (bacterium or fungus) from the blood of an infant who fulfilled all three of the following criteria: (1) One or more of the following infection-related clinical manifestations: respiratory distress, apnea; tachycardia or bradycardia; systemic hypotension or hypoperfusion; hypothermia or fever (T > 38.5°C or <36°C); convulsions, hypotonia, irritability or lethargy; feeding intolerance or intestinal obstruction. (2) One or more abnormal hematologic index: white blood cell count (<5 × 10^9^/L or >30 × 10^9^/L for age ≤3 days or >20 × 10^9^/L for age >3 days), increase of immature/total neutrophil (≥0.16 for age <3 days or ≥0.12 × 10^9^/L for age ≥3 days), C-reactive protein level (≥10 mg/L) or abnormal procalcitonin level (≥0.5 mg/L). (3) Antibiotics used for at least 5 days (11).

Confirmed necrotizing enterocolitis (NEC) was determined using Bell's scale modified by Walsh (12). Briefly, stages Ia–Ib are suspected NEC, stages IIa–IIb are confirmed NEC, and severe/advanced NEC is from stage IIIa with an intact bowel and stage IIIb with a perforated bowel. Confirmed NEC was considered from stage IIa or greater.

Intracranial hemorrhage was defined neonates with subdural, subarachnoid, subependymal, intraventricular, or intraparenchymal hemorrhage (13).

### 2.3. Data collection

Demographic information was obtained from electronic medical records, while clinical data were collected, including gestational age, gender, weight, maternal gestational comorbidities, prenatal and intrapartum conditions. Additionally, laboratory tests were also collected at the onset of the disease (<24 h), including blood count, myocardial enzyme spectrum, coagulation function, C-reactionprotein (CRP), procalcitonin (PCT), serum calcium (Ca), serum sodium (Na), and more. A comparison was conducted between the two groups in terms of duration of oxygen and ventilator use, comorbidities (sepsis, neonatal necrotizing enterocolitis, and intracranial hemorrhage), and outcomes (death or survival).

### 2.4. Statistical analysis

This study uses ARDS as the dependent variable to construct a prediction model, takes the AUC value of the prediction model as the main index, and utilizes PASS 15 software (NCSS, Kaysville, Utah, USA) to calculate the Power of the current sample size. Finally, this study included 79 cases of ARDS and 119 cases of non-ARDS. The AUC value of the prediction model was 0.931. Under the condition of setting two-sided test *α *= 0.05, we inputted data into PASS 15 software (NCSS, Kaysville, Utah, USA) for Curve Tests and obtained a Power >0.999.

Statistical analyses were performed with SPSS Statistics (IBM SPSS Statistics for Windows, Version 26.0. Armonk, NY, US) and R software (Foundation for Statistical Computing, version 4.1.2, Vienna, Austria). Levene's test was used to assess the normality of the measurement data, and those conforming to a normal distribution were presented as mean ± standard deviation ($\bar{X}\pm S$), while the independent samples *t*-test was used for comparison between groups. For nonnormally distributed data, the IQR (interquartile range) was used and the Mann–Whitney *U*-test was used for comparison between groups. Categorical count data were presented as the number of cases (percentage) and group comparisons were made using the *χ*^2^ test. Based on the clinical characteristics of neonatal ARDS in China, easily accessible clinical information was collected, ARDS-related indicators were screened using between-group comparative analysis, multivariate logistic regression analysis was used to calibrate the predictors, and stepwise regression was used to eliminate confounding factors to obtain the prediction model. The area under the curve (AUC) for each index and model was determined using receiver operating curve (ROC), and the *Z*-test was utilized to compare the AUC of each index. The accuracy of the model was internally validated by using a Bootstrap repeated sampling procedure. Moreover, calibration curves were plotted and the concordance index (C-index) of the model was calculated. A level of *P *< 0.05 was considered significant.

## 3. Results

### 3.1. Comparison of general data and laboratory indicators between the two groups

A total of 198 neonates were enrolled in the study, 79 in the ARDS group and 119 in the non-ARDS group. The results of the univariate analysis performed on the two infant cohorts are presented in Table 1. The analysis showed no significant differences between the groups in terms of maternal gestational diabetes, intrahepatic cholestasis, hypertension, maternal placenta previa, premature rupture of membranes, mode of delivery, and laboratory indicators such as postnatal umbilical cord blood arteriovenous PH, Prothrombin Time (PT), Fibrinogen (Fib), and creatine kinase myocardial isoenzyme (CK-MB) (*P *> 0.05); However, there were statistically significant differences in gender, gestational age, weight, maternal prenatal fever, abnormal fetal heartbeat, meconium-stained amniotic fluid (MSAF), white blood cell (WBC), absolute neutrophil count (ANC), neutrophil-percent (NE%), platelet (PLT), PCT, CRP, creatine kinase (CK), activated partial thromboplastin time (APTT), Ca, and S-Na between the two groups (*P *< 0.05).

**Table 1:** Comparison of baseline clinical characteristics and laboratory indicators between the non-ARDS group and ARDS group.

	Non-ARDS group (*n *= 119)	ARDS group (*n *= 79)	*P*
General data			
Gender, male, *n* (%)	71 (59.7)	59 (74.7)	0.029
Gestational age (weeks)	33.30 (31.20, 34.50)	36.60 (33.20, 39.00)	<0.001
Birth weight (kg)	1.87 (1.49, 2.20)	2.67 (1.88, 3.25)	<0.001
Age at admission (days)	3.69 ± 6.12	5.03 ± 5.78	0.126
Age at diagnosis or exclusion of diagnosis (days)	7.58 ± 6.55	9.01 ± 5.24	0.105
Pregnancy complication			
GDM, *n* (%)	40 (33.6)	23 (29.1)	0.506
ICP, *n* (%)	10 (8.4)	3 (3.8)	0.200
PIH, *n* (%)	17 (14.3)	5 (6.3)	0.081
Perinatal condition			
Prenatal fever, *n* (%)	2 (1.7)	7 (8.9)	0.043
Placenta previa, *n* (%)	9 (7.6)	10 (12.7)	0.233
Abnormal fetal heart beat, *n* (%)	2 (1.7)	14 (17.7)	<0.001
Premature rupture of membranes, *n* (%)	35 (29.4)	17 (21.5)	0.217
Mode of production			0.220
Cesarean section, *n* (%)	15 (12.6)	15 (19.0)	
Vaginal delivery, *n* (%)	104 (87.4)	64 (81.0)	
MSAF, *n* (%)	11 (9.2)	24 (30.4)	<0.001
1 min Apgar score	10 (9, 10)	10 (8, 10)	0.004
5 min Apgar score	10 (10, 10)	10 (9,10)	<0.001
10 min Apgar score	10 (10, 10)	10 (10, 10)	0.014
Umbilical vein PH	7.35 (7.29, 7.37)	7.34 (7.26, 7.37)	0.307
Umbilical artery PH	7.31 (7.27, 7.34)	7.32 (7.27, 7.35)	0.609
Laboratory metrics			
WBC (×10^9^/L)	9.56 (6.66,12.23)	12.22 (8.85,18.59)	<0.001
PLT (×10^9^/L)	241.02 ± 64.08	158.20 ± 68.23	<0.001
CRP (mg/dl)	5.00 (5.00,5.00)	5.00 (5.00,5.00)	0.001
ANC (×10^9^/L)	4.98 (3.22, 6.85)	8.29 (4.85, 13.12)	<0.001
NE% (%)	54.90 (46.20,61.50)	64.30 (51.20, 72.30)	<0.001
PT (s)	15.60 (14.10, 18.00)	15.70 (14.30, 17.80)	0.554
Fib (g/L)	1.11 (0.93, 1.41)	1.24 (0.87, 1.77)	0.268
APTT (s)	81.90 (65.90, 98.20)	73.10 (56.10, 86.90)	0.027
Ca (mmol/L)	2.24 (2.13, 2.39)	1.82 (1.37, 2.23)	<0.001
Na (mmol/L)	137.70 ± 2.61	138.63 ± 3.24	0.035
PCT (ng/mL)	0.17 (0.07, 0.73)	0.35 (0.11, 1.27)	0.001
CK-MB (U/L)	143.11 (99.04, 250.50)	133.00 (77.10, 303.43)	0.929
CK (U/L)	284.80 (197.90, 452.80)	411.90 (268.20, 649.90)	0.002

GDM, gestational diabetes mellitus; ICP, intrahepatic cholestasis of pregnancy; PIH, pregnancy induced hypertension syndrome; WBC, white blood cell; PLT, platelet; CRP, C-reactive protein; ANC, absolute neutrophil count; NE%, percentage of neutrophils; PT, prothrombin time; Fib, fibrinogen; APTT, activated partial thromboplastin time; Ca, serum calcium; Na, serum sodium; PCT, Procalcitonin; CK-MB, creatine kinase isoenzyme; CK, creatine kinase.

### 3.2. Neonates with ARDS are more likely to have comorbid sepsis and intracranial hemorrhage and require longer periods of oxygen and ventilator support therapy

Table 2 shows the use of oxygen and ventilators, comorbidities and treatment outcomes for both groups. There was no statistically significant difference between the two groups in terms of NEC and death (*P* > 0.05). However, neonates in the ARDS group were more likely to have comorbid sepsis and intracranial hemorrhage, as well as being dependent on oxygen and ventilators for a significantly longer period of time (*P *< 0.05).

**Table 2:** Treatment and comorbidities in the two groups.

	Non-ARDS group (*n *= 119)	ARDS group (*n *= 79)	*P*
Treatment status			
Oxygen use time (days)	7.00 (5.00, 12.00)	12.00 (6.00, 19.00)	<0.001
Ventilator use time (days)	4.00 (2.00, 7.00)	7.00 (3.00, 14.00)	<0.001
Comorbidities			
Sepsis, *n* (%)	2 (1.7)	12 (15.2)	<0.001
NEC, *n* (%)	6 (5.0)	2 (2.5)	0.610
ICH, *n* (%)	5 (4.2)	21 (26.6)	<0.001
Treatment endings			0.122
Number of deaths, *n* (%)	0 (0.0)	3 (3.8)	
Number of survivors, *n* (%)	119 (100.0)	76 (96.2)	

NEC, necrotizing enterocolitis; ICH, itracranial hemorrhage.

### 3.3. MSAF, increased ANC, and decreased PLT and Ca were independent predictors of neonatal ARDS

The logistic regression analysis employed a forward likelihood approach to incorporate the underlying characteristics, maternal gestational morbidity, maternal pre-natal conditions, maternal conditions at delivery, and early-onset laboratory indicators into the model. The results showed that MSAF, PLT, ANC, and Ca exhibited statistical significance (*P *< 0.05). The final regression model, as shown in Table 3, can be represented by the formula ln (P/1-P) = 9.819 + 1.617 × MSAF - 0.022 × PLT (×10^9^/L) + 0.280 × ANC (×10^9^/L) - 3.935 × Ca (mmol/L). The Hosmer–Lemeshow goodness-of-fit test yielded favorable results, indicating a good model fit.

**Table 3:** Results of multi-factor analysis of independent predictors.

	*B*	SE	Wald*χ*^2^	*P*	OR (95% CI)
MSAF	1.617	0.610	7.018	0.008	5.037 (1.523–16.657)
PLT	−0.022	0.004	30.125	0.000	0.979 (0.971–0.986)
ANC	0.280	0.062	20.419	0.000	1.324 (1.172–1.495)
Ca	−3.935	0.765	26.428	0.000	0.020 (0.004–0.088)
Constants	9.819	1.799	29.785	0.000	–

MSAF: meconium-stained amniotic fluid; PLT: platelet; ANC: absolute neutrophil count; Ca: serum calcium.

### 3.4. High predictive value of the prediction model in neonatal ARDS

**Figure 1:**
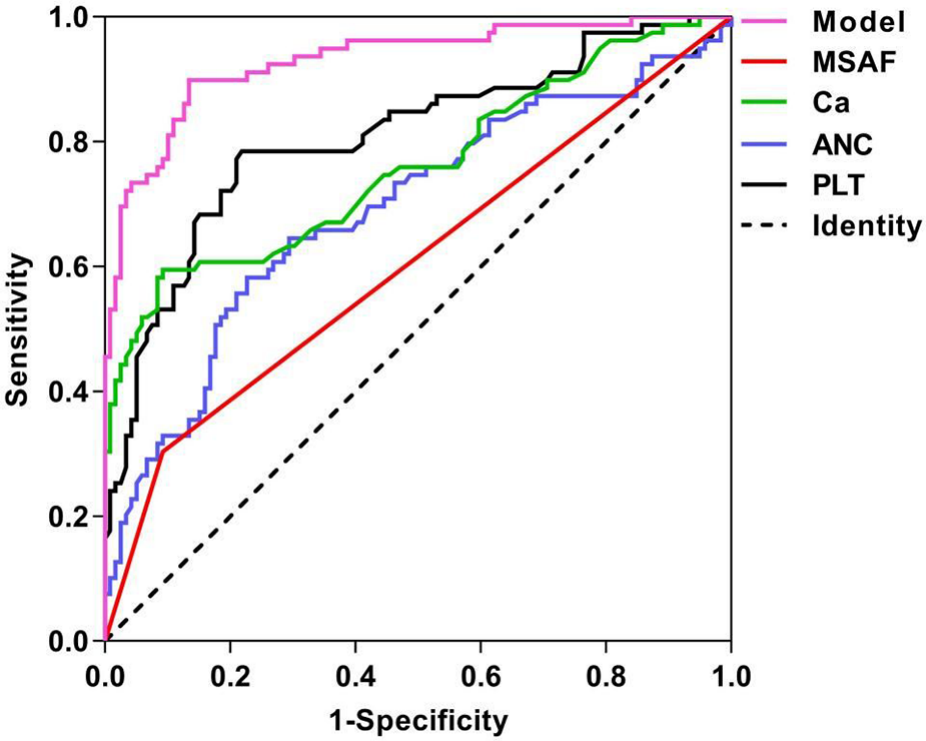
ROC curves for each independent predictor and prediction model of ARDS. MSAF: meconium-stained amniotic fluid; PLT: platelet; ANC: absolute neutrophil count; Ca: serum calcium.

The ROC curves generated for MSAF, PLT, ANC, and Ca and the corresponding AUC values for the prognostic models were 0.606, 0.808, 0.691, 0.761 and 0.931, as illustrated in Figure 1. The cutoff values for MSAF, PLT, ANC, Ca and prognostic models are obtained by employing the maximum of the Jorden index as the optimal threshold, and the associated sensitivity, specificity, positive and negative predictive values are shown in Table 4. The differences in AUC between the combined prognostic model and each independent predictor were statistically significant (*P *< 0.05).

**Table 4:** Comparison of the prediction effects of each independent predictor and prediction model of ARDS.

	AUC	95% CI	*P*	Truncated value	Sensitivity	Specificity	PPV	NPV
MSAF	0.606	0.523–0.688	0.012		30.4	90.8	68.6	66.3
PLT	0.808	0.744–0.873	<0.001	201.50	78.5	78.2	70.5	84.5
ANC	0.691	0.613–0.769	<0.001	7.17	58.2	77.3	63.0	73.6
Ca	0.761	0.689–0.833	<0.001	2.02	59.5	90.8	81.0	77.1
Predictive models	0.931	0.894–0.968	<0.001	0.327	89.9	86.6	81.6	92.8

MSAF: meconium-stained amniotic fluid; PLT: platelet; ANC: absolute neutrophil count; Ca: serum calcium.

**Figure 2:**
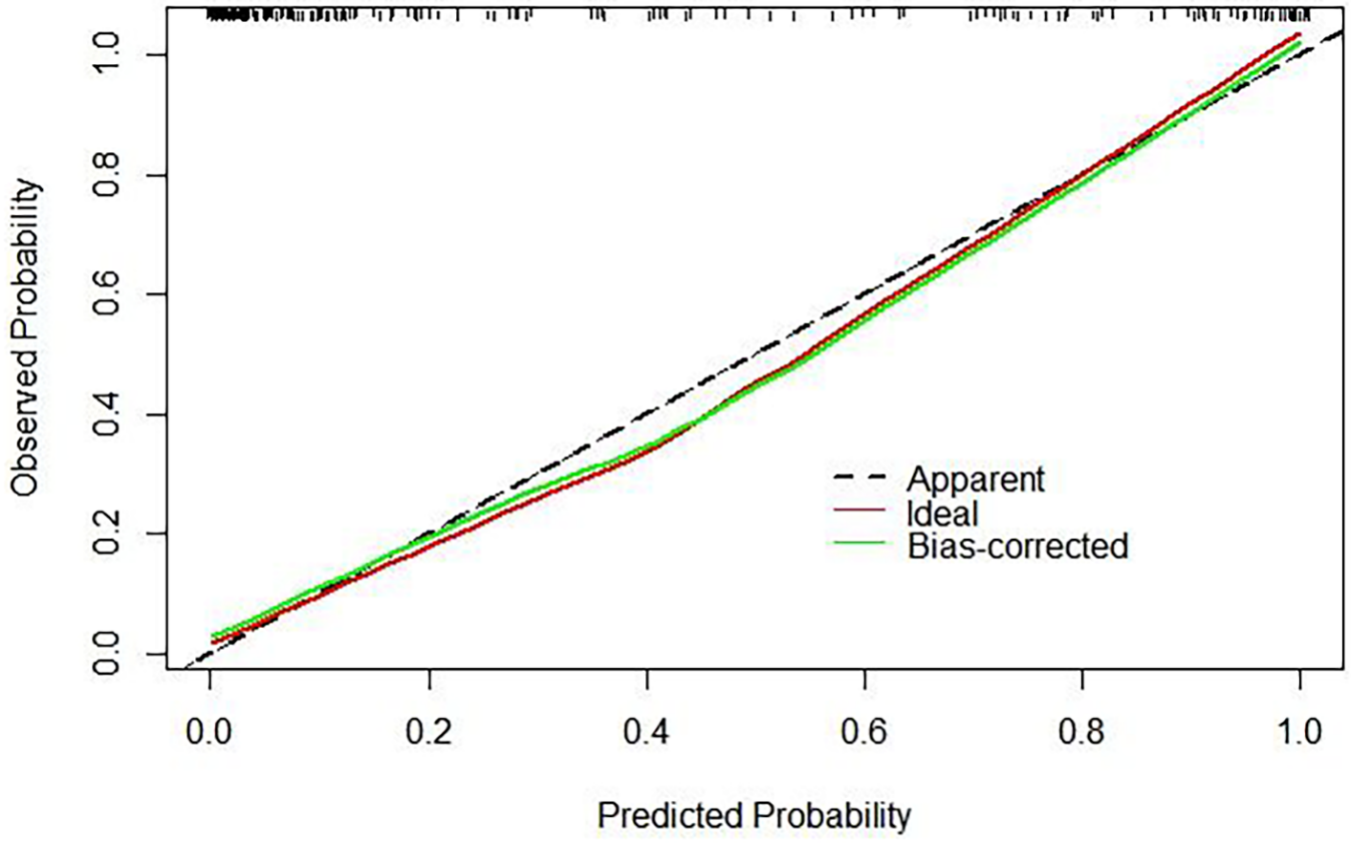
Calibration curve of ARDS model.

By internally validating the accuracy of the prognostic model using the Bootstrap resampling technique, a C-index of 0.931 was obtained, with a strong fit between the original and corrected curves, demonstrating the effectiveness of the prognostic model as shown in Figure 2.

## 4. Discussion

ARDS is a severe pulmonary disease that poses a significant threat to the survival of neonates, particularly preterm infants (10, 14). Due to the intricate etiology and pathogenesis of ARDS, specific treatments remain elusive, making early diagnosis crucial. We found that the presence of MSAF is a significant predictive indicator for ARDS. Approximately 10%–13% of normal pregnancies have fecal contamination of the amniotic fluid, and approximately 4% of infants with this condition may display respiratory distress symptoms (15). MSAF is considered an essential indicator of intrauterine hypoxia (16). In cases of compression of the umbilical cord, parasympathetic activity increases, resulting in elevated vagal tone. This stimulates intestinal peristalsis and relaxation of the anal sphincter, ultimately resulting in the expulsion of fetal fecal matter. Intrauterine hypoxia is a significant risk factor for the development of ARDS. Animal experiments have shown that hypoxia can lead to a decrease in pulmonary phospholipids and lung surfactant proteins, reduced alveolar interstitial vascular density, increased capillary permeability and the formation of hyaline membranes in fetal rats (17). Compensatory wheezing may occur in the fetus under hypoxic conditions, resulting in the aspiration of fecal-stained amniotic fluid into the airway. This direct exposure damages the lung parenchyma and pulmonary vascular endothelium, resulting in capillary leakage, hemorrhagic pulmonary edema, toxic pneumonia, and the progression of respiratory distress (18). Furthermore, inhalation of fecal matter can stimulate the production of a significant quantity of inflammatory mediators, triggering inflammatory cascades within the lungs and the body (19). These mechanisms serve as the underlying pathophysiological basis for the development of ARDS.

Our study shows that ANC can also be used as an indicator for early diagnosis of ARDS in newborns. Neutrophils, the primary cells recruited to the site of inflammation, possess oxidants, proteases, and cationic peptides that give them a potent antimicrobial effect (20). While neutrophil activation is essential for host defense functions, excessive activation can lead to cytotoxicity and the release of immune cell activators such as proteases, cationic peptides, cytokines, and reactive oxygen species, thus resulting in tissue damage (21). Neutrophil activation and migration play a critical role in the progression of ARDS (22, 23). They trigger the formation of neutrophil extracellular traps, produce cytokines such as interleukin-8 (CXCL8), and facilitate the recruitment of different immune cells to modulate the inflammatory process, ultimately culminating in ARDS. CXCL8, which is involved in the recruitment, activation, and degranulation of neutrophils, amplifies inflammation and contributes to disease progression (24). Studies have demonstrated a correlation between the number of neutrophils in bronchoalveolar fluid and the severity of ARDS in patients with ARDS (25, 26). Animal experiments have revealed that reducing neutrophil levels mitigates the severity of lung injury in mice (27). Additionally, Juss et al. (28) observed that peripheral blood polymorphonuclear leukocytes from ARDS patients exhibited an activated state and abnormal oxidative stress responses.

Platelets contain pre-formed molecules within their internal granules that are released upon platelet activation along with several inflammatory mediators, including IL-1β (29–31). Our study shows a decrease in platelet count during the early stages of neonatal ARDS. Observations in peripheral veins have demonstrated platelet rolling along intact vascular endothelium. Notably, this activity is significantly enhanced in the presence of inflammatory stimuli and endothelial cell stress. Subsequently, platelets adhere to the vascular endothelium by a fibrinogen-dependent mechanism facilitated by the intercellular adhesion molecules ICAM-1 and platelet GP2b/3a (32), contributing to a decrease in circulating platelet levels. Platelets may also exert a significant influence on neutrophil-mediated lung injury through synergistic interactions (33), as they interact directly with both neutrophils and monocytes and serve as a notable source of pro-inflammatory cytokines. Neutrophils integrate signals from the alveolar endothelium, potentially establishing a link between the vessel wall and platelets, ultimately leading to vascular injury.

Our research also suggests that low serum calcium (Ca) is a prognostic factor for early neonatal ARDS. Hypocalcemia primarily manifests in critically ill patients with sepsis and burns (34), and it has been suggested that elevated levels of circulating pro-inflammatory cytokines disrupt systemic calcium homeostasis (35). Animal studies have shown significant reductions in serum parathyroid hormone (PTH), 1,25 (OH)2D and calcium following intraperitoneal injection of IL-1β or IL-6 (36).

WBC, PCT, and CRP serve as markers associated with inflammation. One study revealed a significantly higher prevalence of ARDS in cardiac surgery patients with elevated PCT levels, suggesting that elevated PCT levels may serve as an early indicator of ARDS in postoperative cardiac surgery patients (37). Hoeboer SH et al. (38) discovered that CRP has some predictive value and is useful in monitoring the severity of ARDS in intensive care units. Although univariate analysis in this study identified some predictive value of WBC, PCT, and CRP in predicting ARDS, the final regression prediction model did not incorporate multiple factors, likely due to the relatively limited sample size.

According to the International Multicenter Study of Neonatal ARDS (1), sepsis, meconium aspiration, and neonatal pneumonia are the most important causes of neonatal ARDS in that order especially meconium aspiration, are more commonly observed in late preterm or term infants, which explains the findings of our study: the gestational age of neonate in the ARDS group was greater than that in the non-ARDS group.

Accurate determination of disease progression during the early stages of ARDS, coupled with prompt, targeted treatment of affected neonates, may improve treatment efficiency, reduce complications, and shorten the duration of treatment. Thus, the importance of early prediction models in the clinical setting is obvious. Unfortunately, there is currently no specific early prediction method available for neonatal ARDS. In our study, we gathered clinically accessible data, utilized multivariate logistic regression analysis to refine predictive factors, and employed MSAF, PLT, ANC, and Ca levels to construct an early diagnostic model for neonatal ARDS. This approach effectively mitigated the lack of specificity associated with individual indicators. Notably, the combined model showed superior predictive power compared to individual predictors. We internally validated the predictive ability of the model and it performed satisfactorily.

## 5. Limitations

This study is a retrospective, single-center investigation with a relatively limited sample size. Although the model underwent internal validation, additional prospective studies are needed to substantiate and confirm the clinical efficacy of the model. Furthermore, this study was conducted specifically on neonates presenting respiratory distress as the primary clinical symptom and may not be applicable to neonates with other symptoms as the main clinical manifestation or to healthy neonates.

## 6. Conclusion

In summary, this study established a prediction model for neonatal ARDS based on the clinical characteristics of neonatal ARDS and collected clinically easily accessible data. However, to comprehensively validate these findings, a multicenter prospective study is indispensable.

## Data Availability

The raw data supporting the conclusions of this article will be made available by the authors, without undue reservation.
